# Simulation of electrochemical behavior in Lithium ion battery during discharge process

**DOI:** 10.1371/journal.pone.0189757

**Published:** 2018-01-02

**Authors:** Yong Chen, Weiwei Huo, Muyi Lin, Li Zhao

**Affiliations:** 1 Beijing Information Science and Technology University, Collaborative Innovation Center of Electric Vehicles in Beijing, Beijing, China; 2 State Key Laboratory of Automotive Safety and Energy, Tsinghua University, Beijing, China; Chongqing University, CHINA

## Abstract

An electrochemical Lithium ion battery model was built taking into account the electrochemical reactions. The polarization was divided into parts which were related to the solid phase and the electrolyte mass transport of species, and the electrochemical reactions. The influence factors on battery polarization were studied, including the active material particle radius and the electrolyte salt concentration. The results showed that diffusion polarization exist in the positive and negative electrodes, and diffusion polarization increase with the conducting of the discharge process. The physicochemical parameters of the Lithium ion battery had the huge effect on cell voltage via polarization. The simulation data show that the polarization voltage has close relationship with active material particle size, discharging rate and ambient temperature.

## 1. Introduction

The Lithium-ion battery has become one of the most widely used energy storage devices because of its high energy and power densities [1–12]. However, their applications are limited by the performance limitations. But a higher performance will result in serious polarization which is caused by mass transport limitations within the electrolyte and electrodes for the same type of battery. The impact for each process to the polarization is dependent on the dynamic and kinetic material properties, the battery design and the charging-discharging mechanism. The relationship between the influencing factors and the polarization are very complex. So it is necessary to build a mathematical model to understand the polarization phenomenon in details.

Numerical simulation based on mathematical models is an effective method to study the relationship between the corresponding parameters and battery performance. An equivalent circuit model was established by Nyman *et al*. [13] for studying the polarization effect on cell voltage. The modified hybrid pulse power characterization test was taken for identifying the polarization resistance. Kim *et al*. [14] presented an equivalent circuit models to represent the electrochemical properties to predict the discharging procedure. The battery parameters such as the thickness of separator, the thickness of electrode and the electrolyte concentration were changed to study their influence on charging procedure. The results showed that charging time could be affected by adjusting the thickness of the cathode [15]. Normally, electrochemical models are based on chemical/electrochemical kinetics and tansport equations to simulate the Li-ion batteries’ reaction [16]. T.R. Ashwin [17] proposed a Pseudo-Two-Dimensional porous electrode model to predict the battery pack’s overall behavior. The individual cell in the pack develop uniform SEI resistance indicating that the pack is stable without significant split current variation. Zhang *et al*. [18] estimated a numerical method to describe the influence factors on the diffusion polarization by numerical simulation. The result showed that there should be a positive correlation between the diffusion polarization and the active material size at a certain range. The same relation exists between the diffusion polarization and the thickness of the electrodes. The polarization characteristics were investigated qualitatively with the methods mentioned above. Some researchers pay attention to increasing the battery performance and decreasing the polarization voltage by changing methods and battery components. A degradation model for Li-ion batteries was developed considering side reactions [19]. The analysis had revealed that the capacity fade was predominantly caused by loss of ions and active materials. Yang *et al*. [20] presented a Li-ion battery model coupled electrochemical-thermal. The electrochemical reactions and heat transfer had been added to the model. The results showed that polarization voltage was negatively related with charging performance and positively related with heat generation. Yan *et al*. [21] built a half cell model with reconstructed realistic LiCoO_2_ cathode electrode. The results showed that the polarization due to ionic transport depend on the position and porosity. Some researchers proposed a battery model using 3D microstructure for detailed studies [22–29]. However, qualitatively analysis on polarization voltage and its relationship with discharging performance by building an electrochemical model were not covered.

In this work, we have chosen to an electrochemical model and simulate a ternary battery during a discharging test. The model has high accuracy to make a prediction about the distributed electrical behaviour on discharging procedure. The influencing factors of the solid phase diffusion polarization and the liquid phase diffusion polarization of the electrode have been studied and some suggestions for reducing the diffusion polarization has been proposed. This work would facilitate to optimizing the battery design by providing the theoretical support and shorting the development cycle. It may promote a low-cost method and make a positive contribution to manufacturing Li-ion batteries for large-scale applications.

## 2. Model and experimental

### 2.1 Model description

**Fig 1:**
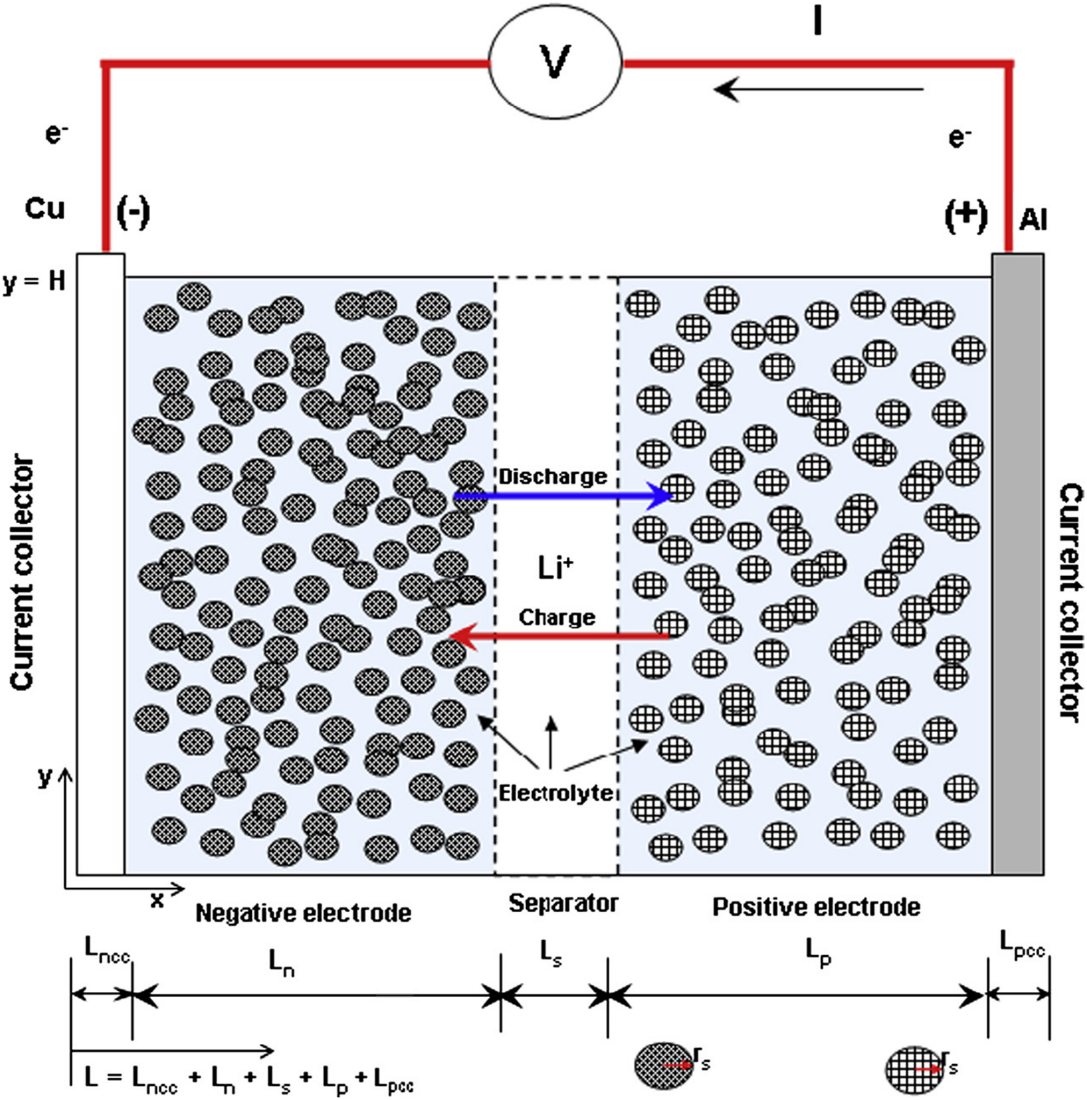
Schematic of lithium ion battery electrochemical model.

The Fig 1 showed an electrochemical model for the Li-ion battery. The model was set as a sandwich structure, including two current collectors, the positive electrode, the negative electrode and the separator. The electrodes are set as porous that consists of active particles with spherical shapes of uniform sizes.

A model describing the electrochemical behaviour of the Li-ion battery cell was set up and solved in COMSOL Multiphysics with the parameters presented in Tables 1–4. The model constitutes a theoretical basis for the discussion regarding the polarization arising in the battery. Newman is accredited for the development of the general mathematical methodology of the full battery model and similar models have been published previously [30–32].

**Table 1:** 

Nomenclature	
*a*_*s*_	Specific interfacial area, m^2^m^-3^
*c*	Concentration of the binary electrolyte, mol m^-3^
*D*	Diffusion coefficient of lithium ion, m^2^ s^-1^
*F*	Faraday constant, 96487 C mol^-1^
*I*_*app*_	Applied current density, A m^-2^
*J*	Pore wall flux of lithium ions, mol m^-2^s^-1^
*k*	Electrochemical reaction rate constant, m^2.5^ mol^-0.5^s^-1^
*L*	Thickness of battery component, m
*R*	Gas constant, 8.3145 mol^-1^ K^-1^
*R*_*s*_	Radius of electrode particle, m
*t*	Time, s
*T*	Temperature, K
*t*_*+*_	Transference number of lithium ion
*U*	Open-circuit potential, V
*x*	Spatial coordinate
*ε*	porosity
*ε*_*f*_	Volume fraction of fillers
*ρ*	Density, kg m^-3^
*δ*_*i*_	Electronic conductivity of solid matrix, S m^-1^
*Φ*	Potential in the phase, V
Subscripts	
eff	effective
max	maximum
n	Negative electrode
p	Positive electrode
s	Solid phase
e	electrolyte
sep	separator

**Table 2:** Equations for the electrochemical model.

Name of equation	Expression of equation
Ohm’s law	$-\sigma_{\text{s}}^{\text{eff}}\frac{\partial \Phi_{\text{s}}}{\partial x}=i_{\text{s}}$ (1)
Charge conservation, solid	$-\frac{\partial i_{\text{s}}}{\partial x}=\sigma_{\text{s}}^{\text{eff}}\frac{\partial^{2}\Phi_{\text{s}}}{\partial x^{2}}=aFj$ (2)
Charge conservation, liquid	$\frac{\partial }{\partial x}\left(\sigma_{\text{e}}^{\text{eff}}\frac{\partial \Phi_{\text{e}}}{\partial x}\right)=-aFj+\frac{2RT(1-t_{+}^{0})}{F}\frac{\partial }{\partial x}\left(\sigma_{\text{e}}^{\text{eff}}\frac{\partial \text{ln}c_{\text{e}}}{\partial x}\right)$ (3)
Substance conservation, solid	$\frac{\partial c_{s}}{\partial t}=\frac{1}{r^{2}}\frac{\partial }{\partial r}\left(D_{\text{s}}^{}r^{2}\frac{\partial c_{s}}{\partial r}\right)$ (4)
Substance conservation, liquid	$\varepsilon_{\text{e}}\frac{\partial c_{\text{e}}}{\partial t}=\frac{\partial }{\partial x}\left(D_{\text{e}}^{\text{eff}}\frac{\partial c_{\text{e}}}{\partial x}\right)+(1-t_{\text{+}}^{\text{0}})aj$ (5)
Butler-Volmer equation	$j=i_{0}\cdot [\text{exp}(\frac{\alpha_{n}F}{RT}\eta_{s})-\text{exp}(-\frac{\alpha_{p}F}{RT}\eta_{s})]$ (6)
Reaction over potential	$\eta_{s}=\Phi_{s}-\Phi_{\text{e}}-U-j\cdot R_{SEI}$ (7)
Exchange current density	$i_{0}=k\cdot {\left(c_{\text{e}}\right)}^{\alpha_{n}}{\left(c_{\text{s},\text{max}}-c_{\text{s},e}\right)}^{\alpha_{n}}{\left(c_{\text{s,e}}\right)}^{\alpha_{p}}$ (8)
SOC in the particle	$y=\frac{3}{R_{s}^{3}}\int_{0}^{R_{s}}r^{2}\frac{c_{\text{s}}}{c_{\text{s},\text{max}}}dr$ (9)
Potential changes with temperature	$U=U_{\text{ref}}(y)-(T-T_{\text{ref}})\left[\frac{dU}{dT}\right]$ (10)

**Table 3:** The variables in the electrochemical model.

Symbol	Description	Unit
Φ_s_	Electrical potential in solid phase	V
Φ_e_	Electrical potential in electrolyte phase	V
*c*_s_	Particle concentration in solid phase	mol/m^3^
*c*_e_	Salt concentration in electrolyte phase	mol/m^3^
*j*	Molar flux	mol/m^2^∙s
*i*_*s*_	Electrode current density	A/m^2^

**Table 4:** The physical parameters in the electrochemical model.

Symbol	Unit	Value		
*i*_1C_	A/m^2^	15		
*F*	A∙s/mol	96485		
*$t_{\text{+}}^{\text{0}}$*	1	0.363		
Symbol	Unit	Negative	Separator	Positive
*L*	m	120×10^−6^	25×10^−6^	110×10^−6^
$\sigma_{\text{s}}^{\text{eff}}$	S/m	*$\varepsilon_{\text{s}}^{1.5}\sigma_{\text{s}}$*		*$\varepsilon_{\text{s}}^{1.5}\sigma_{\text{s}}$*
*σ*_s_	S/m	100×*g*(*T*)		100×*g*(*T*)
*ε*_s_	1	0.471		0.297
*$\sigma_{\text{e}}^{\text{eff}}$*	S/m	$\varepsilon_{\text{e}}^{1.5}\sigma_{\text{e}}$	*σ*_e_	$\varepsilon_{\text{e}}^{1.5}\sigma_{\text{e}}$
*σ*_e_	S/m	*σ*_e_ = *f*(*c*_*e*_)	*σ*_e_ = *f*(*c*_*e*_)	*σ*_e_ = *f*(*c*_*e*_)
*ε*_e_	1	0.357		0.444
*D*_s_	m^2^/s	1.5×10^−13^×*h*(*T*)		5×10^−13^×*g*(*T*)
$D_{\text{e}}^{\text{eff}}$	m^2^/s	$\varepsilon_{\text{e}}^{1.5}D_{\text{e}}$	*D*_e_	$\varepsilon_{\text{e}}^{1.5}D_{\text{e}}$
*D*_e_	m^2^/s	7.5×10^−11^	7.5×10^−11^	7.5×10^−11^
*R*_*s*_	m	4×10^−6^		2×10^−6^
*A*	1/m	3*ε*_s_ / *R*_s_		3*ε*_s_ / *R*_s_
*α*_*n*_, *α*_*p*_	1	0.5		0.5
*R*_*SEI*_	Ω∙m^2^	0.001		0.001
*K*	m/s	2×10^−11^		2×10^−11^
*c*_s,max_	mol/m^3^	21,000		20,000
*Y*	1	0.80		0.09
*U*_ref_	V	Fig 1		Fig 1
*$\frac{dU}{dT}$*	V/K	-0.00005		-0.0001

It is supposed that the positive electrode consists three phases: an electronically conductive phase, an active material phase, and a liquid electrolyte phase. Electrons are transported in the conductive phase between the current collector and the active material. The variation in solid phase potential in the conductive phase is described by the Ohm’s law. The related model has been built in [33].

### 2.2 Experiment setup

**Fig 2:**
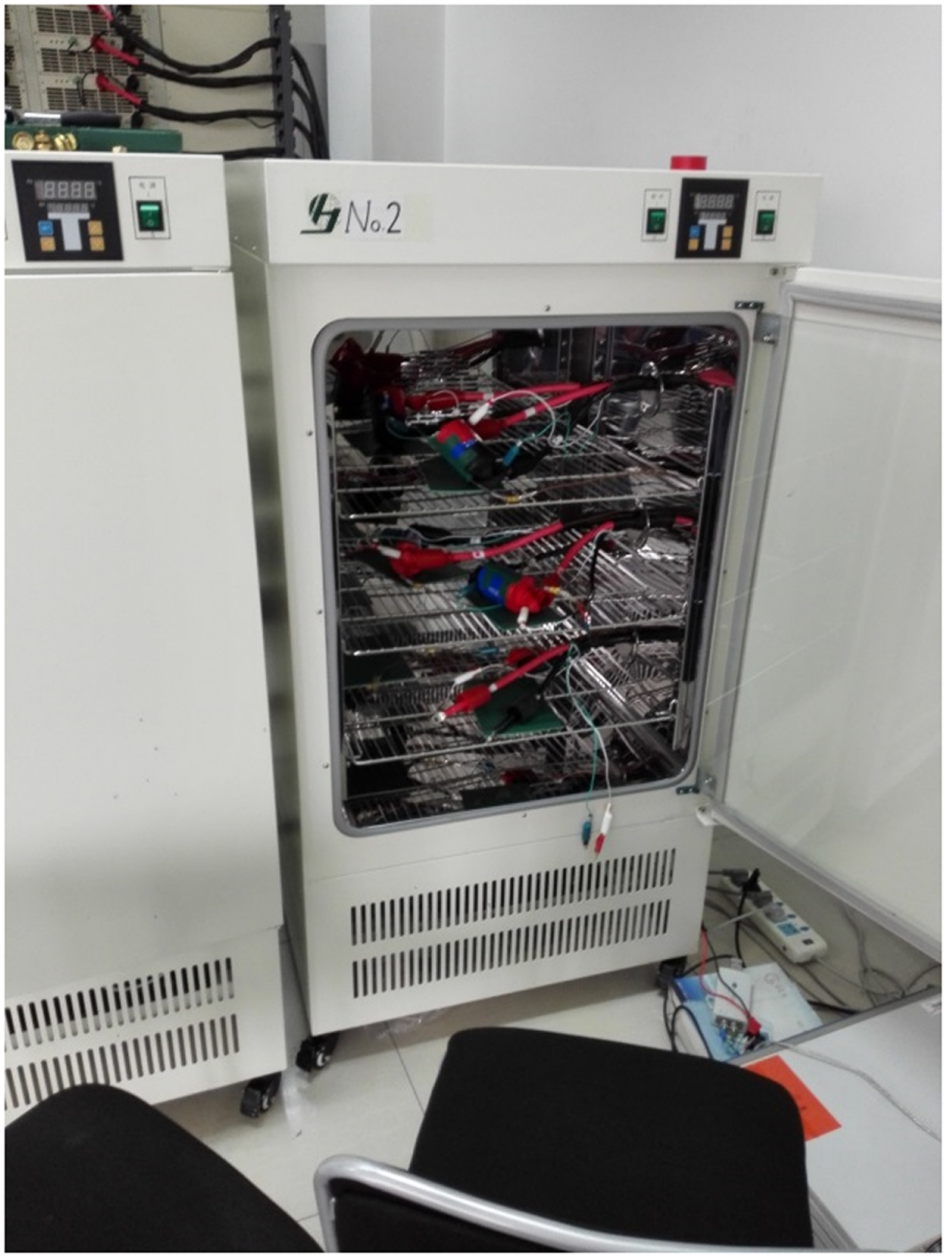
The experiment scene of the battery discharging.

A commercial soft-packing battery was tested, which the capacity was 7.8 Ah, and the nominal voltage was 3.6 V. The battery was charged/discharged using an Arbin BT2000 cycler, the ambient temperature was set using a thermal chamber (YINHE thermal chamber). The Arbin BT 2000 cycler is used to collect data including the working voltage and charge-discharge current. The experiment scene could be shown in Fig 2.

The measurement divides into two parts: (1) reference performance test (RPT). The maximum capacity for the battery could be got at this procedure. (2) charging-discharging test. The battery was charged and discharged at different current rates at different ambient temperatures.

The RPT is fixed under the temperature of 298K. The charging current is set 1/3C and the discharging current is set 1C. The maximum available capacity of the battery would be confirmed if the capacity deviation for the three test results is within 2%

The charging-discharging tests for the battery are set at the following procedure. The charging procedure is the same as that in RPT. However, the discharging process is conducted at different current rates (1/3C, 1C. 2C) under variant temperatures (283K, 298K, 308K).

## 3. Results and discussion

### 3.1 Model validation

The discharging curves of experimental data and simulated results have been compared to validate the accuracy of the model.

**Fig 3:**
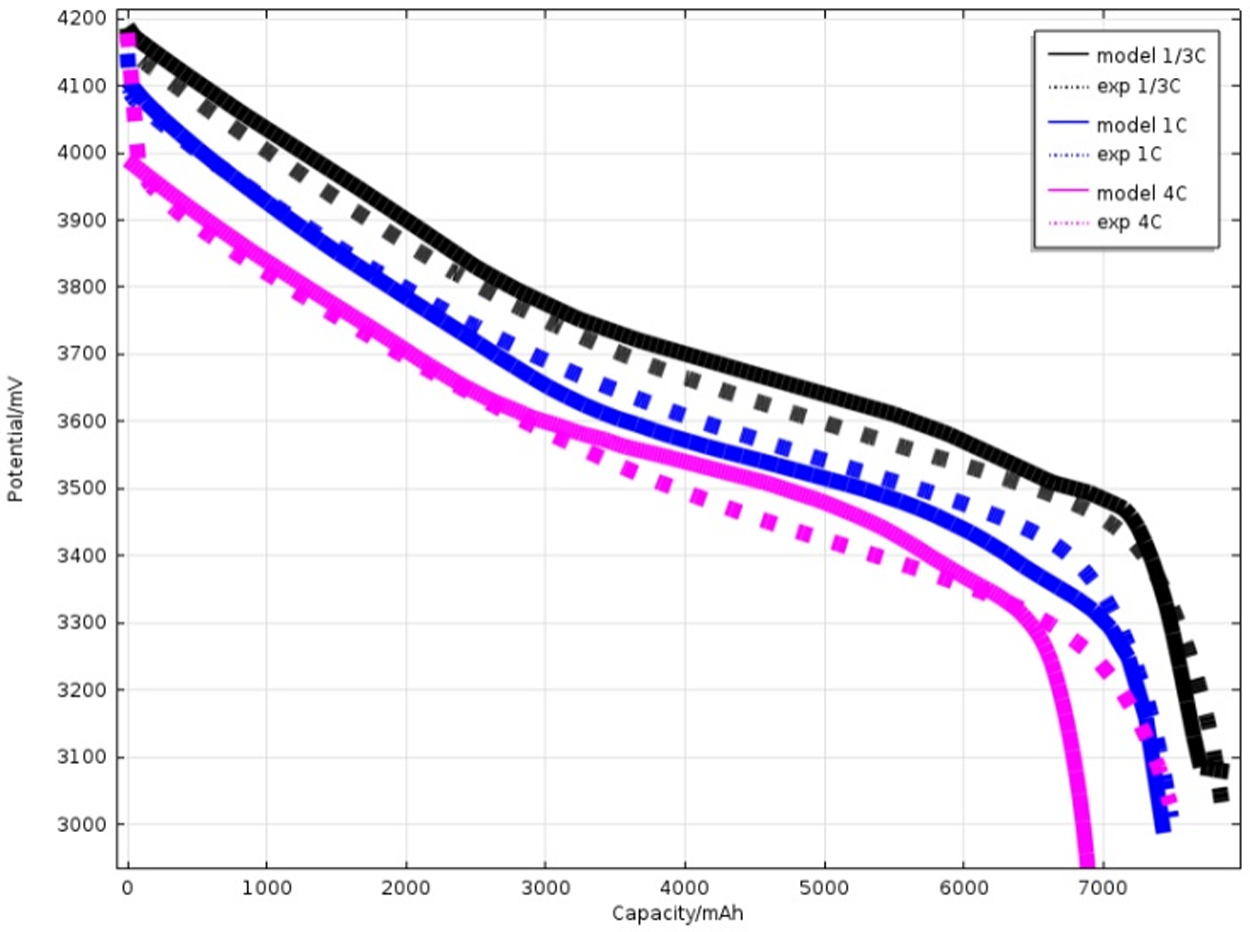
Comparison of simulated results of working voltage with experimental data during different discharge rate (1/3 C, 1C, and 4 C) under 25°C.

As shown in Fig 3, the discharging curves based on simulation and experiment has been compared at different discharging rate under ambient temperature. The discharging capacity is 7.868 Ah, 7.491 Ah and 7.482 Ah with the experimentally measured (The discharging rate is 1/3 C, 1 C and 4 C respectively). And the discharging capacity is 7.924 Ah, 7.473 Ah and 6.912 Ah by simulation(The discharging rate is 1/3 C, 1 C and 4 C respectively). The results show that the modelling curves are overlapped the experimental data basically at the whole discharge cycle. The discharge plateau and discharge capacity are presented with different discharge rate. A little inconformity is occurred at the end of the discharging curves. According to the Fig 3, the discharge voltage plateau is higher at a lower discharging rate. The reaction rate at the electrode surface is slower than the electron accumulation rate at a higher discharging rate. The accumulated electron which could not to be consumed will result in the electric chemical polarization potential and lead to the lower battery open circuit voltage.

**Fig 4:**
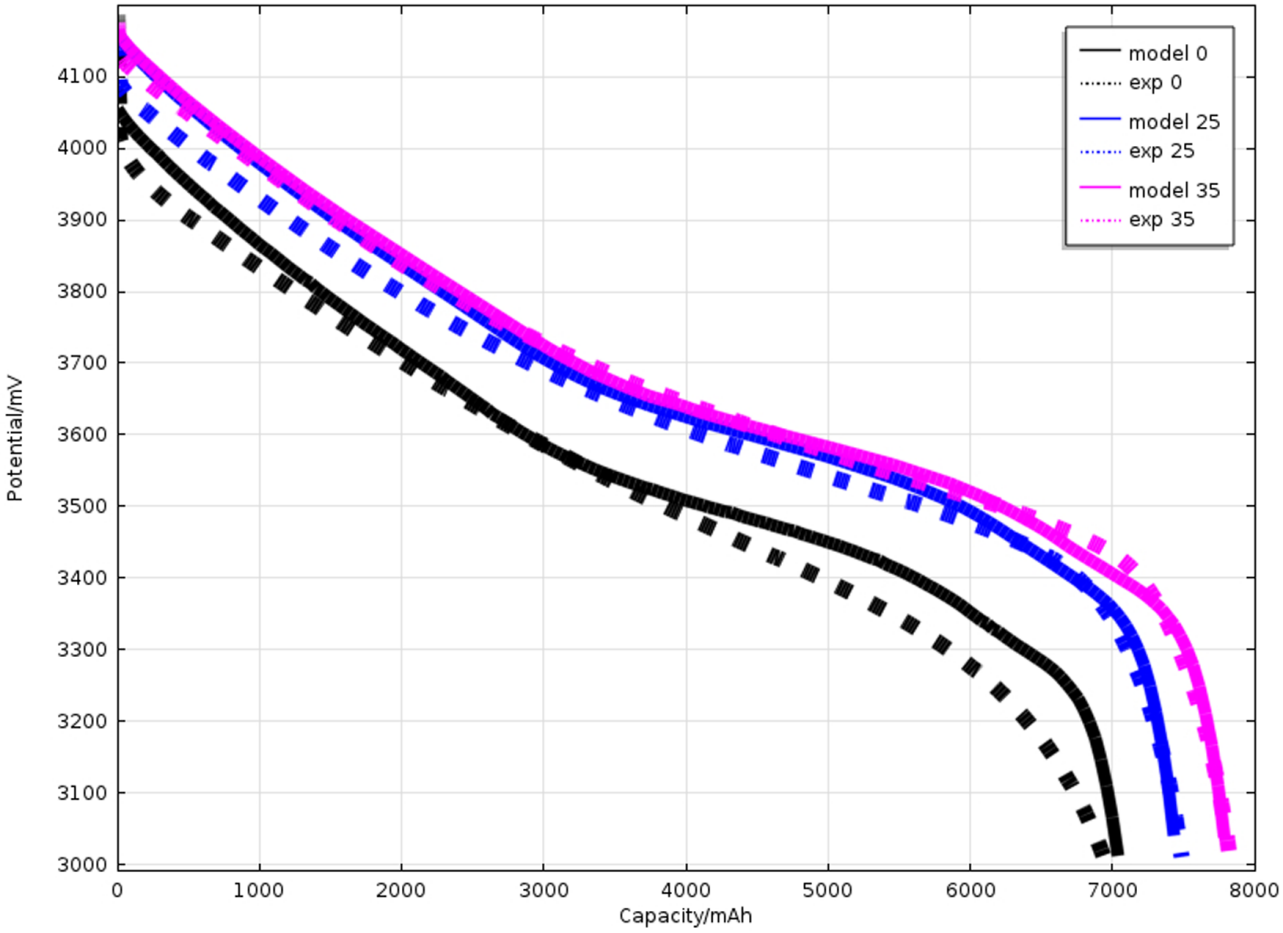
Comparison of simulated results of working voltage with experimental data during different ambient temperature (0°C, 25°C, and 35°C) under 1C discharge rate.

Fig 4 shows the data getting from the simulation and experiment at different temperature at a constant discharge rate (1 C). The discharging capacity is 6.943 Ah, 7.491 Ah and 7.831 Ah with the experimentally measured (The temperature is 0°C, 25°C and 35°C respectively). And the discharging capacity is 7.024 Ah, 7.503 Ah and 7.829 Ah by simulation (The temperature is 0°C, 25°C and 35°C respectively). The compare results present that discharging curves have the same trend with a little deviation. The discharge voltage plateau is higher at 25°C. The higher temperature means a higher electrochemical reaction velocity, a lower ohm resistance. If the temperature is much higher, the electric chemical polarization will dominate the main role gradually. The potential will be reduced on the contrary.

**Fig 5:**
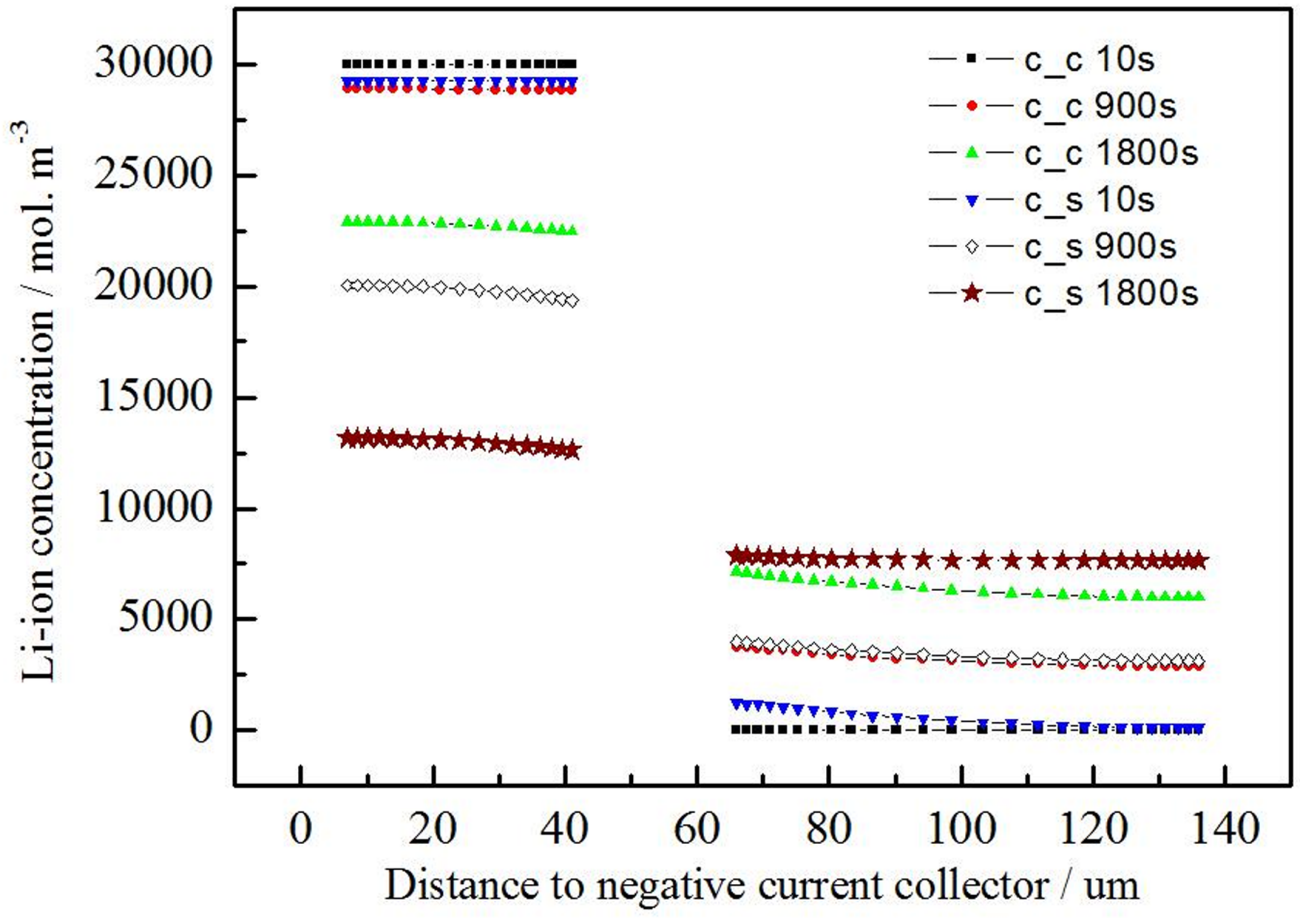
Li ion concentration at center (c_c) and surface (c_s) of active material particles across electrode with different discharging time (0s, 900s and 1800s) at 1C discharging rate.

Fig 5 shows that distribution of the active material particles at the center and surface position of the electrodes and the surface of the lithium ion concentration. The Li ion concentration difference exists in the thickness direction for the electrode. The electrode is the porous structure, and the electrolyte is filled in the gap of the electrode. The Li ion transport to the active material particle surface through the electrolyte. The active material takes part in the reaction near the surface of the electrode first, resulting in a liquid phase diffusion polarization. Further, the active material concentration difference of the negative electrode is less than the positive electrode.

For describe the diffusion situation clearly, the maximum, minimum and average Li ion concentration was simulated at the surface of electrode with different shelve time at Table 5. The concentration difference is reduced with the shelve time increasing. Furthermore, the reduced rate of the negative electrode is less than the one of the positive electrode. The maximum concentration difference of the negative electrode is fell to 70.39% than the origin value when the shelve time is 200S, the average concentration is 5% reduced than the same situation. And the corresponding values is 12% and 1.12% for the positive electrode.

**Table 5:** The maximum, minimum and average Li-ion concentration at the surface of electrode with different shelve time.

Shelve time (s)	Negative electrode (mol/m^3^)				Positive electrode (mol/m^3^)			
	Cavg	Cmax	Cmin	ΔCmax	Cavg	Cmax	Cmin	ΔCmax
0	6946.4	7152.6	6740.2	412.3	11770	12333	11206	1127
100	8044.9	8207.0	7882.8	324.2	11440	11594	11286	308
200	8269.3	8414.4	8123.8	290.6	11426	11494	11358	136

The result shows that the liquid phase diffusion polarization for the positive electrode is less than the diffusion polarization for the negative electrode. According to the Table 4, the difference of electrolyte volume fraction for the two electrodes is small. The Li ion diffusion coefficient is equal for the two electrodes at this model. So the length of the electrode is a main influencing factor for the liquid phase diffusion polarization.

### 3.2 Polarization influence factor analysis

The influence factor for the polarization voltage includes the reaction process and mass transport. Therefore, the factors which have relationship with the reaction and mass transport should be paid more attention to.

The porous electrode structure has a certain thickness, since the position of the respective infiltration of the electrolyte varies, the density difference can be formed and the impact of the electrode / solution interface electrochemical reaction rate when the electrolyte concentration inside the Li ion battery received. Therefore, the concentration of there is a difference would cause electrochemical reaction rate inconsistencies.

**Fig 6:**
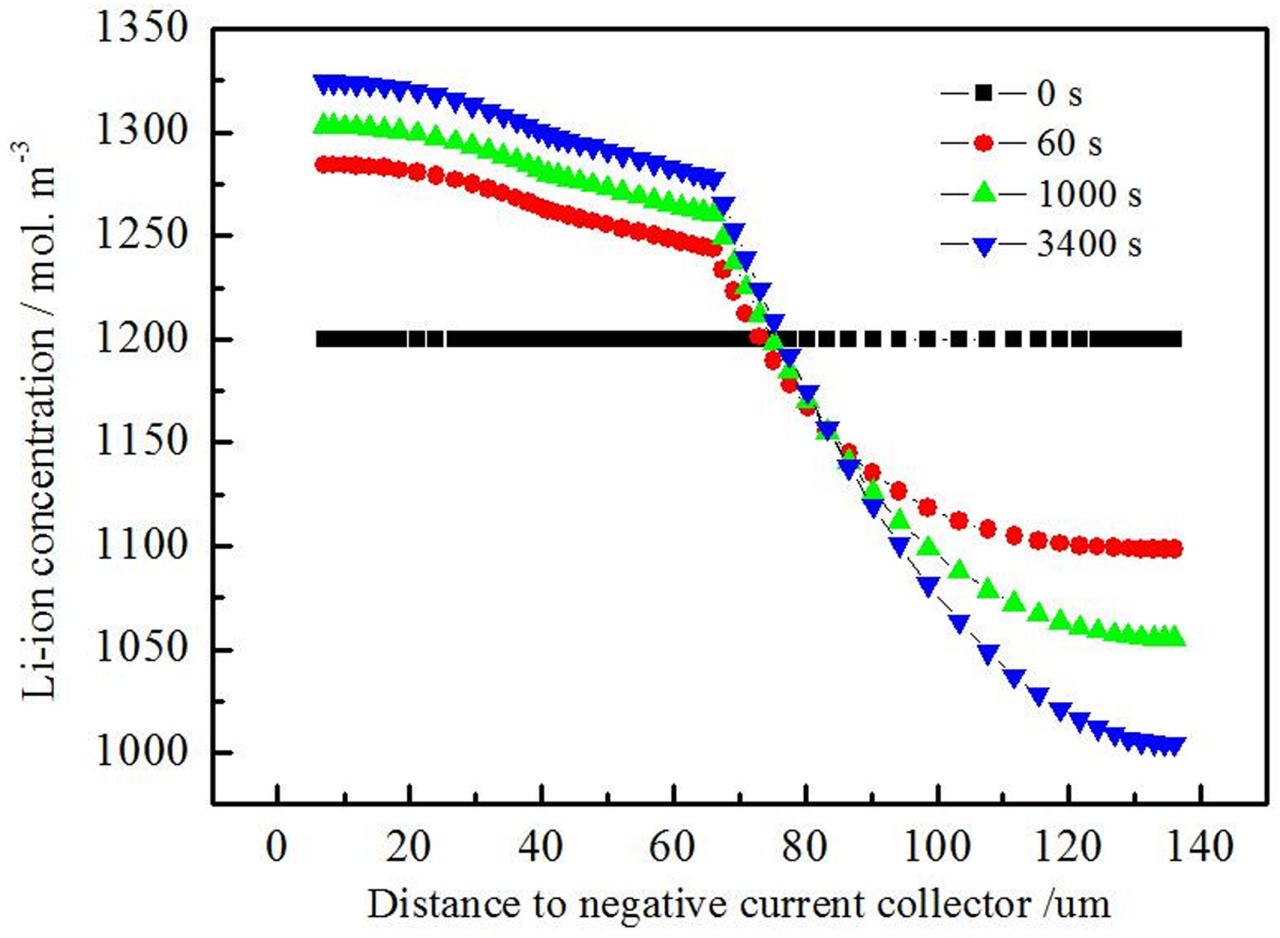
Distribution of electrolyte salt concentration for the 1C discharging.

Simulation results from Fig 6 show that electrolyte salt concentration for the discharging at 1C. The distribution of the electrolyte salt concentration is uniform at the beginning of the discharging procedure, and the negative active material is in Li-rich state. Li ion releases from porous electrode material into the electrolyte with the discharging procedure. As a result, the electrolyte concentration around positive electrode decreases and the electrolyte concentration around negative electrode increases with the discharging procedure. And the further from the separator, the more serious of the concentration deviation from the initial value.

**Fig 7:**
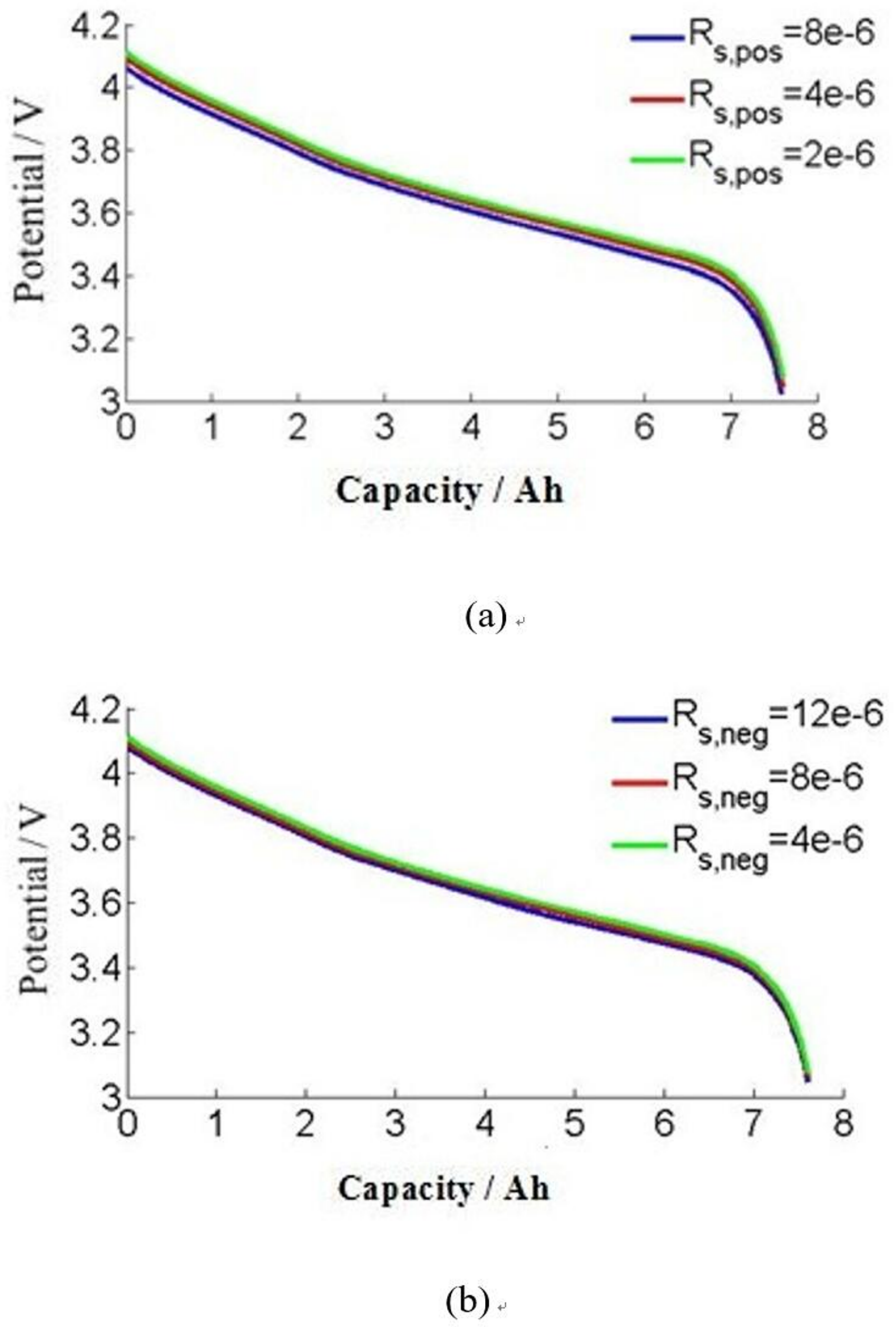
Comparison of simulated results of working voltage during different particle radius. (a) positive electrode, (b) negative electrode.

Fig 7 shows that discharging curve with different particle radius. The particle radius is 2 μm, 4 μm and 8 μm for positive electrode. The particle radius is 4 μm, 8 μm and 12 μm for the negative electrode. The capacity is little reduced with the particle radius changed. The total of Li ion has little relationship with the particle radius for this model. But the discharging potential is increasing a little at the beginning of the curve, with the radius is decreasing. As the negative electrode for example, the Li ion is much easier to dis-insertion when the negative particle radius is half of the original. And the specific area is larger with the reducing of the radius. It is helpful to improve the electrochemical reaction rate, reduce the activation polarization, and decrease the polarization potential.

**Fig 8:**
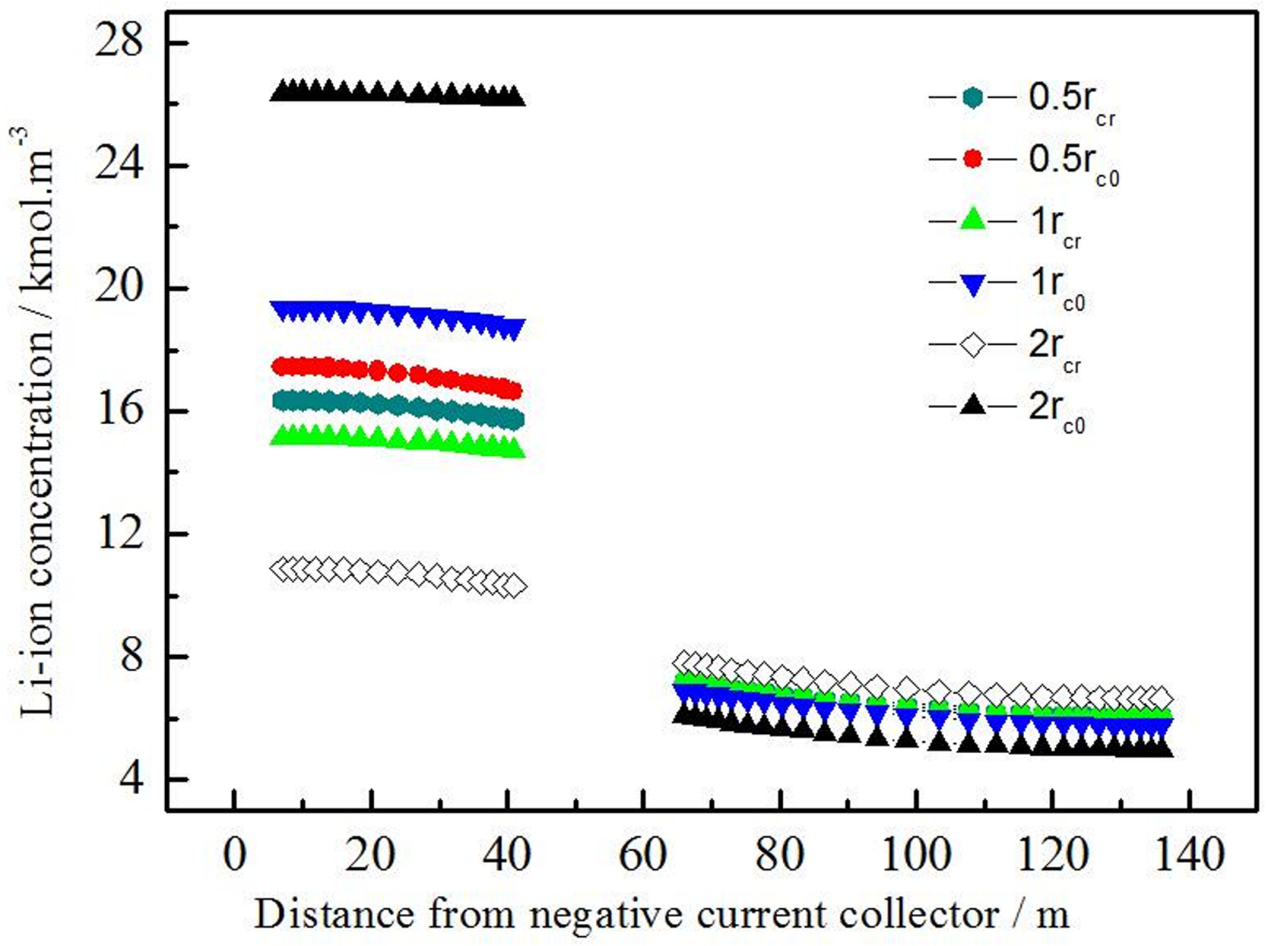
Li-ion concentration at center (c_0_) and surface (c_r_) of active material particles across electrode with different active material particle sizes.

Fig 8 shows the Li ion concentration at center (c_0_) and surface (c_r_) of active material particles across electrode with different active material particle sizes discharged at 1C for 1800s. The concentration difference of Li-ion is significantly reduced when the active material particles is half of the original (26.07% reduced in negative electrode, 25.35% reduced in positive electrode). On the contrary, the concentration difference of Li-ion is significantly increased when the active material particles is twice larger than the original (278.46% increased in negative electrode, 386.83% increased in positive electrode).

It is showed that decreasing the particle size of the active material can reduce the solid phase diffusion polarization effectively. That may be the smaller particle size of Li ion, the shorter of insertion and dis-insertion distance. With the same diffusion coefficient, the smaller particle means the shorter time to balance the Li-ion concentration on the surface and the center. A larger specific area means a lower surface current density at the same discharging current density, resulting in decreasing the diffusion polarization for solid phase.

**Fig 9:**
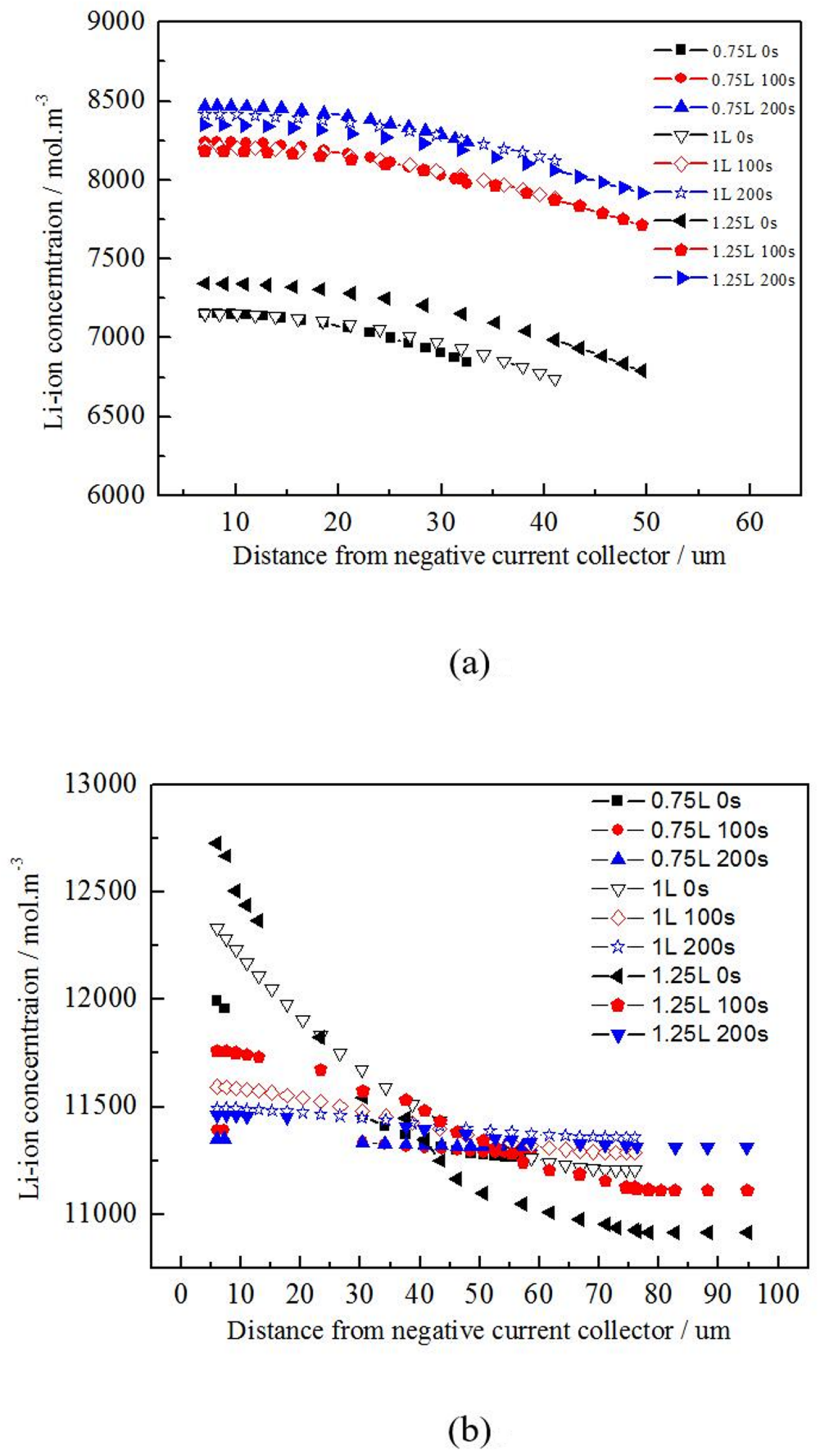
Li-ion concentration at surface of negative active particles across electrode of batteries with different electrode thickness. (a) negative electrode, (b) positive electrode.

Fig 9 shows the Li ion concentration at surface negative active material particles across electrode of batteries with different electrode thickness relaxed 0, 100, 200 s after discharging at 1C for 1800 s. With the same ratio of active material and same discharging rate, the concentration difference is positive correlation with the electrode thickness. According to the Fig 7, the Li ion concentration is gradual changed. The smaller for ratio of Li ion concentration difference and average concentration is, the more uniform for Li ion distribution will be. The thickness of electrode will change the liquid phase diffusion path. Reducing the thickness of electrode properly is helpful for reducing the liquid phase diffusion polarization.

According to the Table 6, the uniformity of the Li concentration has closely relationship with the electrode thickness in the direction of electrode. The Li ion concentration difference has positive correlation with the thickness of electrode. The path of the liquid diffusion will be changed with the thickness variation of electrode. And the time which the Li ion transports through the electrolyte to the internal will be changed with at the same diffusion coefficient. Therefore, decreasing the thickness of the electrode at a certain range will be benefit to reducing the liquid phase diffusion polarization.

**Table 6:** The Li-ion concentration at surface of negative active particles across electrode of batteries with different electrode thickness relaxed 0, 100, 200s after discharging 1C for 1800s.

Shelve time (s)	Negative (ΔCmax/Cavg)			Positive (ΔCmax/Cavg)		
	0.75L	1L	1.25L	0.75L	1L	1.25L
0	4.39%	5.89%	7.74%	6.32%	9.71%	13.52%
100	3.23%	4.01%	5.87%	0.95%	2.70%	6.43%
200	2.77%	3.50%	5.26%	0.33%	1.19%	3.47%

## 4. Conclusions

The electrochemical model for Li-ion battery is built to study the influence factors for polarization characteristics by changing the value of the parameters.

During the battery discharging process, the electrochemical reaction rate is different at the different positions of the electrodes, resulting in the electrochemical polarization. At the beginning of the discharging, the reaction rate near the membrane is highest, and the reaction rate near the collection is lowest. With the time of discharging process, the reaction rate near the collector is increased gradually and the reaction rate near the membrane is decreased gradually. Similarly with the electrochemical reaction rate, the mass non-uniformity can also cause the diffusion polarization. The solid and liquid phase diffusion polarization is more and more serious with the discharging process. The size of active material particle has a great influence on solid phase diffusion. The thickness of electrode is a main factor for affecting the liquid phase polarization.

## Supporting information

S1 File The data for Fig 5. Li ion concentration at center (c_c) and surface (c_s) of active material particles across electrode with different discharging time (0s, 900s and 1800s) at 1C discharging rate.
(OPJ)

S2 File The data from the Figs 3 to 9. (ZIP)
